# Supplementary material for: Regulation of A-to-I RNA editing and stop codon recoding to control selenoprotein expression during skeletal myogenesis
Source: Nat Commun. 2022 May 6;13:2503. doi: 10.1038/s41467-022-30181-2 (PMC9076623; doi:10.1038/s41467-022-30181-2)
Supplement: Supplementary file 1 — Supplementary Information [file 41467_2022_30181_MOESM1_ESM.pdf]

Supplementary information

Regulation of A-to-I RNA editing and stop codon recoding to control selenoprotein expression during skeletal myogenesis

Yuta Noda et al.

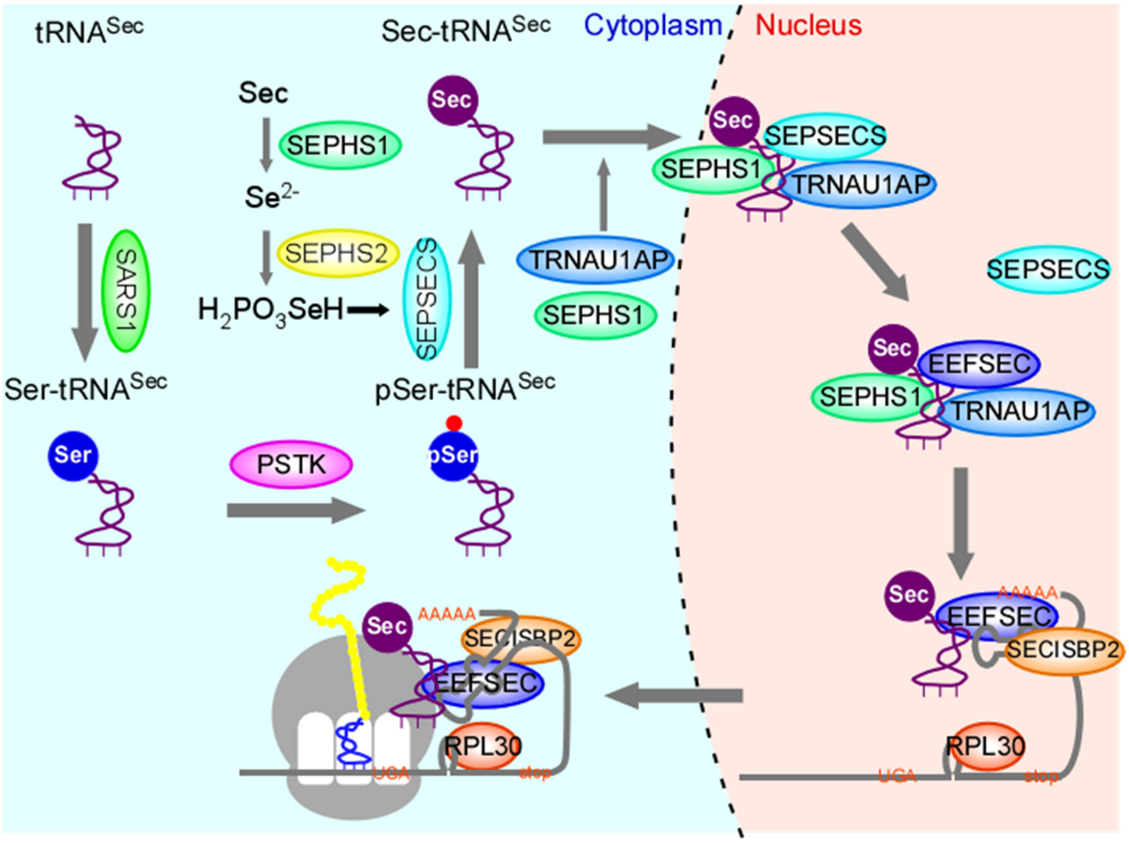

Supplementary Figure 1. Schematic illustration of the UGA/Sec recoding machinery in eukaryotes.

Ser and Sec attached to $tRNA^{Sec}$ are shown as blue and purple circles, respectively. The small red circle on the blue circle represents the phosphate group of phosphoserine.

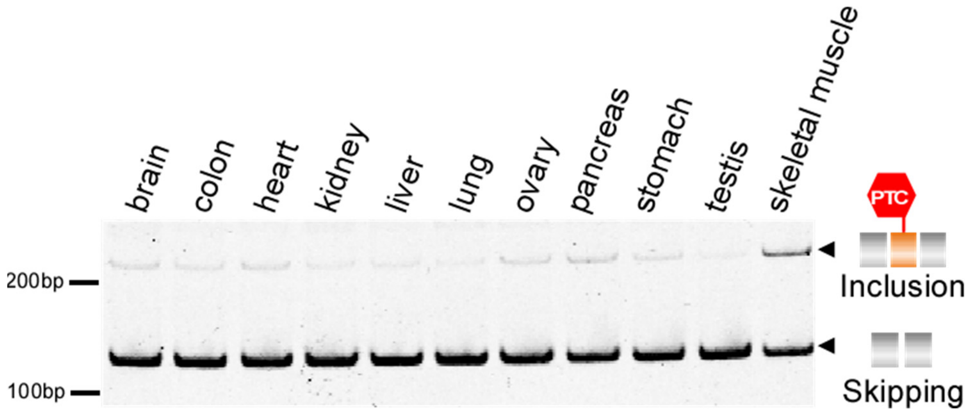

Supplementary Figure 2. Alu exonization of *SELENON* mRNA in various human tissues.

cDNAs between exons 2 and 3 of *SELENON* mRNA in various human tissues were amplified by RT-PCR. The inclusion and skipping isoforms are indicated schematically on the right. This experiment was performed once. Unprocessed gel image is provided in Source Data file.

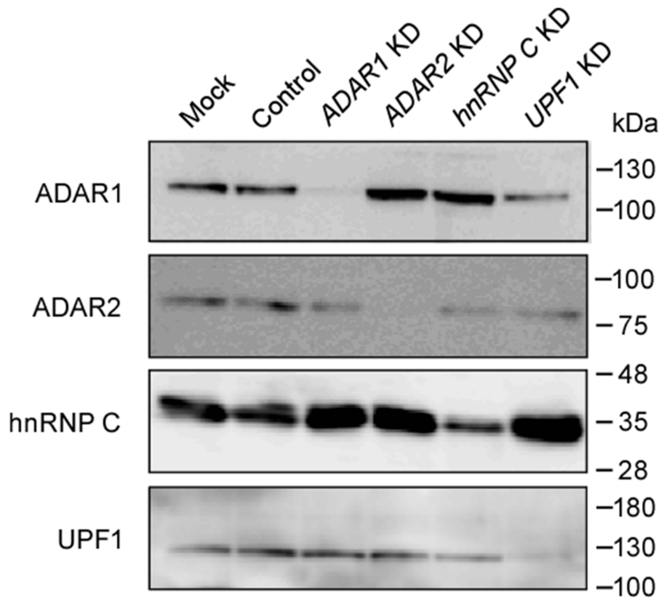

Supplementary Figure 3. RNAi-mediated knockdown of *ADAR1*, *ADAR2*, *hnRNP C*, and *UPF1* in HeLa cells.

Validation of the knockdown experiment (Figure 1d) by western blotting. This experiment was performed once. Unprocessed gel images are provided in Source Data file.

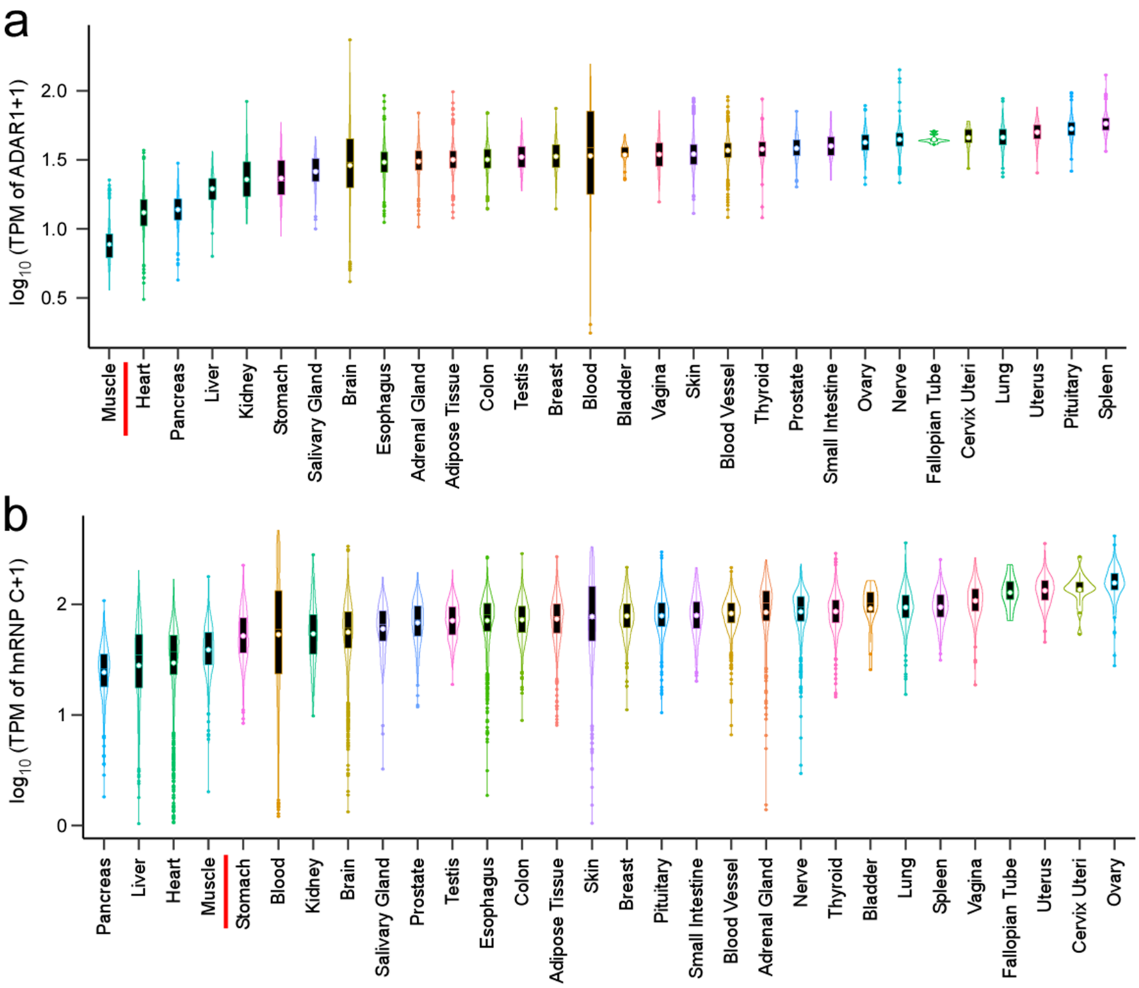

Supplementary Figure 4. Expression profiles of *ADAR1* and *hnRNP C* in human tissues.

Log_{10} (transcripts per million [TPM] of *ADAR1*+1) (a) and Log_{10} (TPM of *hnRNP C*+1) (b) from various human tissues were obtained from GTEx Analysis V8 [1], and depicted as violin and box plots. For box plot, open circles indicate median, box limits indicate upper and lower quartiles, whiskers indicate 1.5 × interquartile range and points indicate outliers. Mean values in each tissue are indicated by white circles; *n*=1204 (Adipose Tissue), 258 (Adrenal Gland), 21 (Bladder), 929 (Blood), 1335 (Blood Vessel), 2642 (Brain), 459 (Breast), 19 (Cervix Uteri), 779 (Colon), 1445 (Esophagus), 9 (Fallopian Tube), 861 (Heart), 89 (Kidney), 226 (Liver), 578 (Lung), 803 (Muscle), 619 (Nerve), 180 (Ovary), 328 (Pancreas), 283 (Pituitary), 245 (Prostate), 162 (Salivary Gland), 1809 (Skin), 187 (Small Intestine), 241 (Spleen), 359 (Stomach), 361 (Testis), 653 (Thyroid), 142 (Uterus) and 156 (Vagina) individuals. Source data are provided in Source Data file.

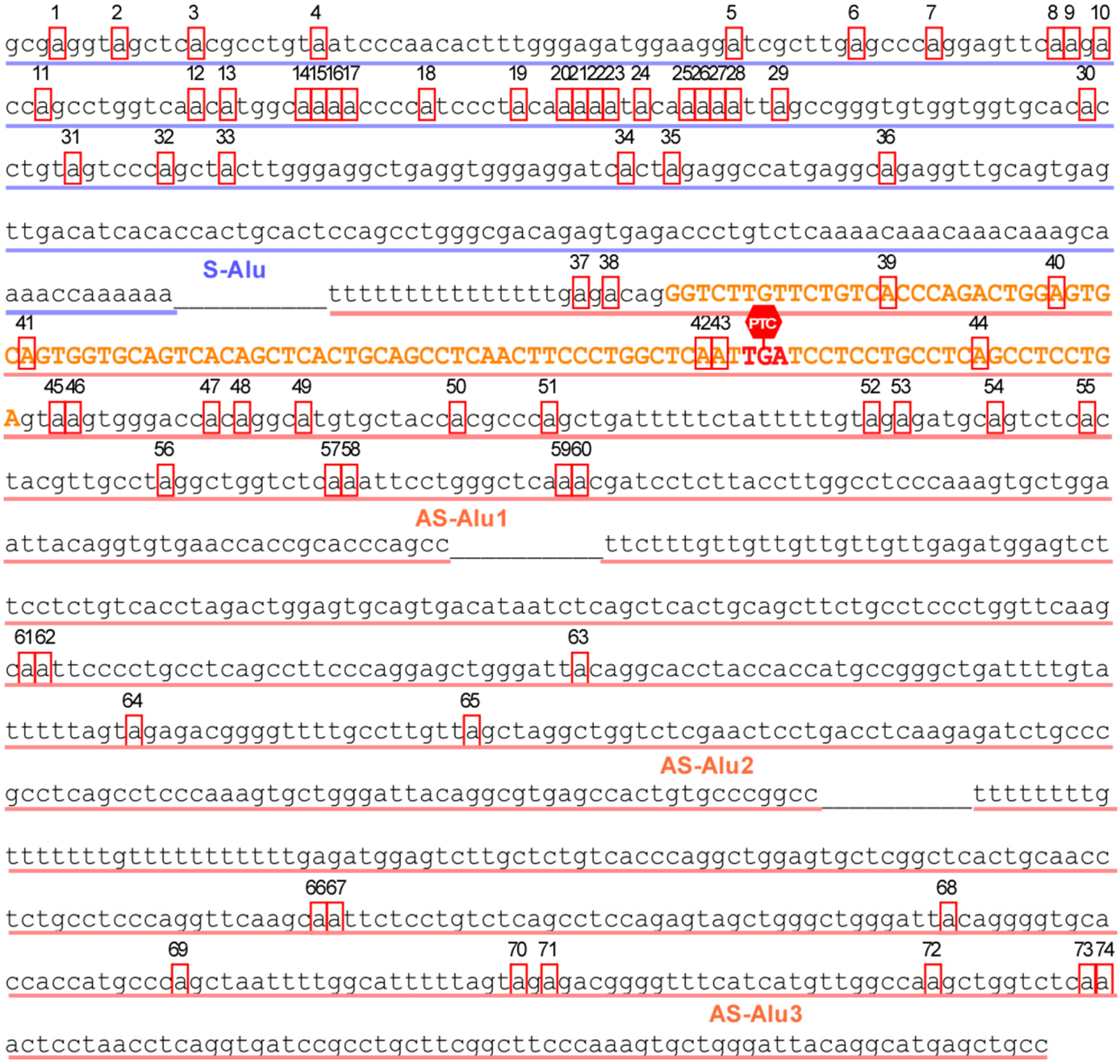

Supplementary Figure 5. Alu sequences in intron 2 of the human *SELENON* gene. The A-to-I RNA editing sites mapped in this study are boxed in red, with the site numbers shown above. The regions of S-Alu and AS-Alu1,2,3 are underlined in blue and red, respectively. Nucleotide sequence of the Alu exon is shown in orange uppercase text. The TGA of the PTC is shown in red.

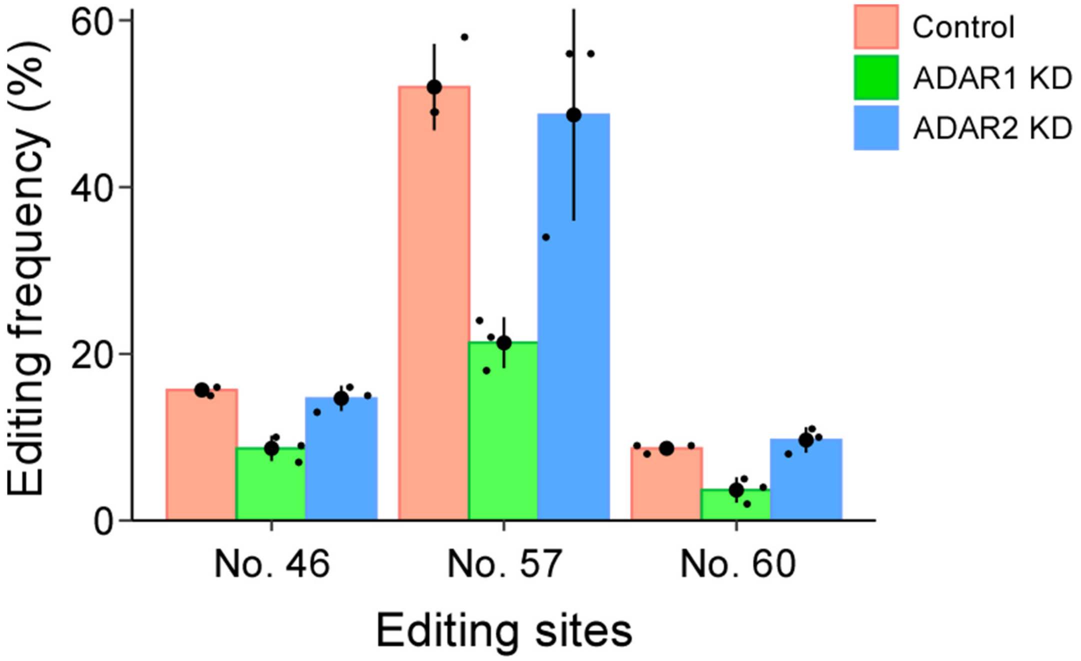

Supplementary Figure 6. A-to-I RNA editing in AS-Alu1 upon knockdown of *ADAR1* or *ADAR2*.

Frequencies of the editing sites No. 46, 57, and 60 of *SELENON* mRNAs in HeLa cells were measured by calculating the peak height ratios of A and G in the Sanger sequencing chromatogram of cDNAs [2]. HeLa cells were treated with siRNAs targeting luciferase (control), *ADAR1*, and *ADAR2*. Data are presented as mean values +/- S.D; *n*=3 biologically independent samples. Source data are provided in Source Data file.

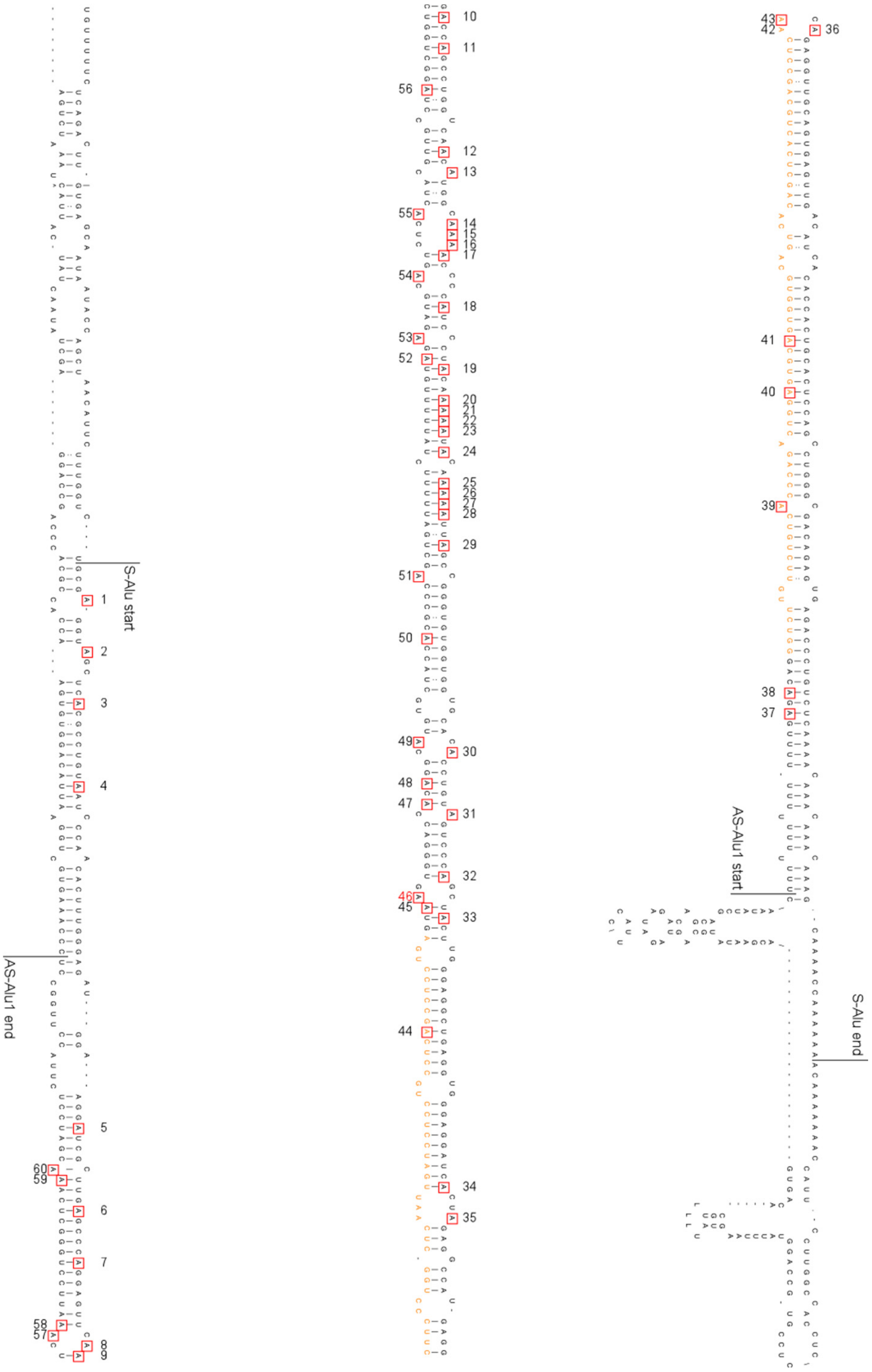

Supplementary Figure 7. A-to-I RNA editing sites mapped in double-stranded structures formed by S-Alu and AS-Alu1 of *SELENON*.

Mfold [3] prediction of the secondary structures of S-Alu and AS-Alu1 of *SELENON* mRNA. The A-to-I RNA editing sites mapped in this study are boxed in red, with the site numbers. The Alu exon is shown in orange.

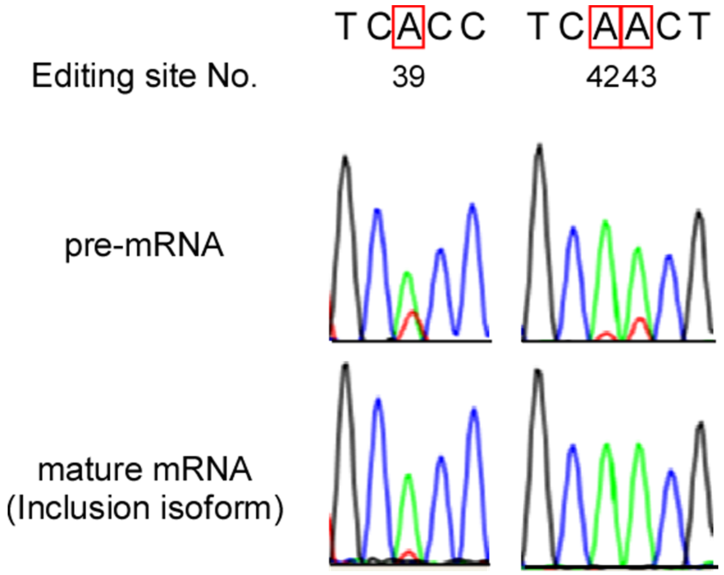

Supplementary Figure 8. cDNA sequences of the Alu exon in the precursor and mature mRNAs of *SELENON*.

cDNAs of pre- (upper panel) and mature (lower panel) mRNA were amplified from human brain total RNAs. A-to-I RNA editing sites are boxed in red, with the site numbers shown below. The electropherograms for A, G, T, and C are shown in green, red, gray, and blue, respectively.

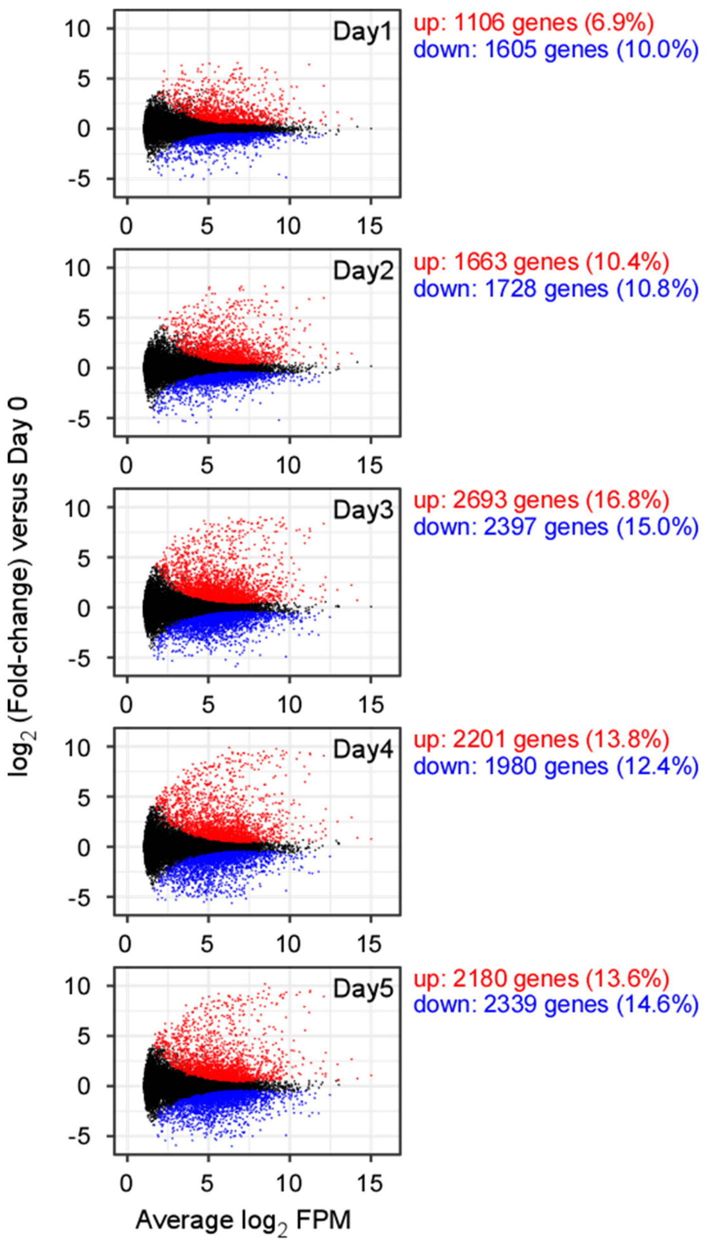

Supplementary Figure 9. Transcriptome analysis of Hu5/KD3 differentiation. Differential expression of Hu5/KD3 cells on each day of differentiation, normalized against expression on Day 0. The $\log_2$(fold-change) of each gene was plotted against the average $\log_2$(FPM). Significantly upregulated and downregulated genes (FDR $<$ 0.01) are highlighted in red and blue, respectively. Source data are provided in Source Data file.

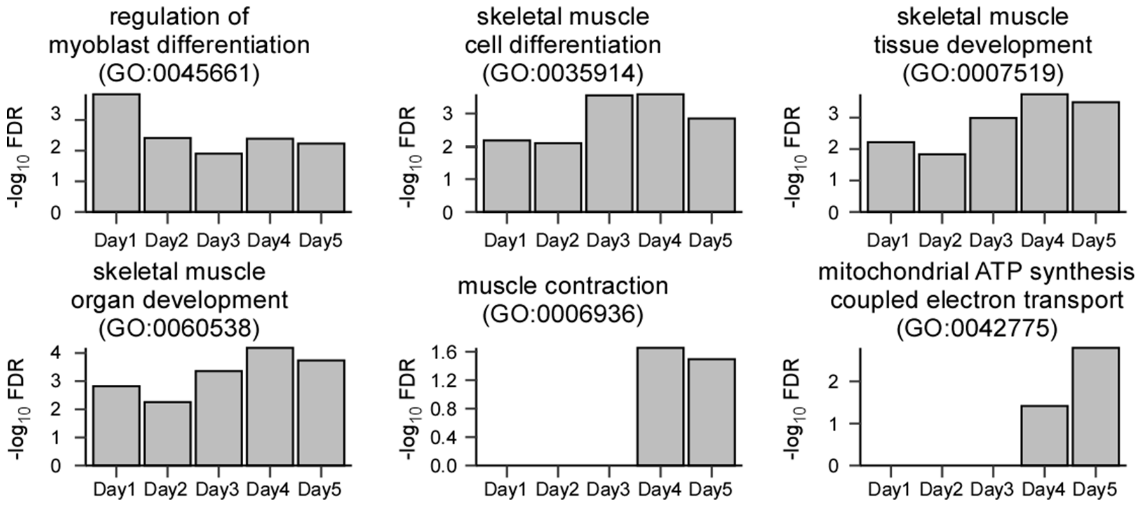

Supplementary Figure 10. Gene ontology analysis of differentially expressed genes during Hu5/KD3 differentiation.

The $-\log_{10}$(FDR) of selected GO terms between each day of differentiation versus Day 0 calculated using a PANTHER overrepresentation test and Bonferroni correction with FDR<0.01. Source data are provided in Source Data file.

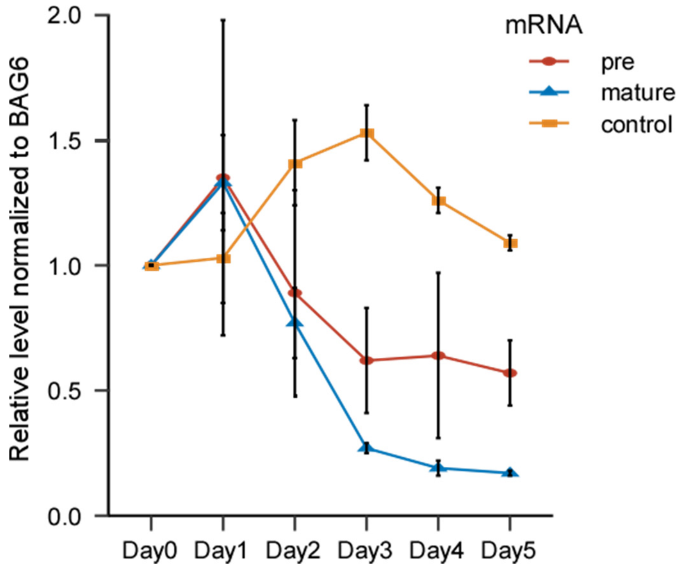

Supplementary Figure 11. RT-qPCR analysis to quantify pre-mRNA and mature mRNA of *SELENON*.

Relative levels of precursor (circle) and mature (triangle) mRNAs of *SELENON,* measured by RT-qPCR of total RNA from each day of Hu5/KD3 differentiation. *FKBP1A* mRNA (square) was used as a control. All data were normalized against *BAG6* mRNA because its steady-state level did not change in the RNA-seq data. Data are presented as mean values +/- S.D; *n*=3 biologically independent samples. Source data are provided in Source Data file.

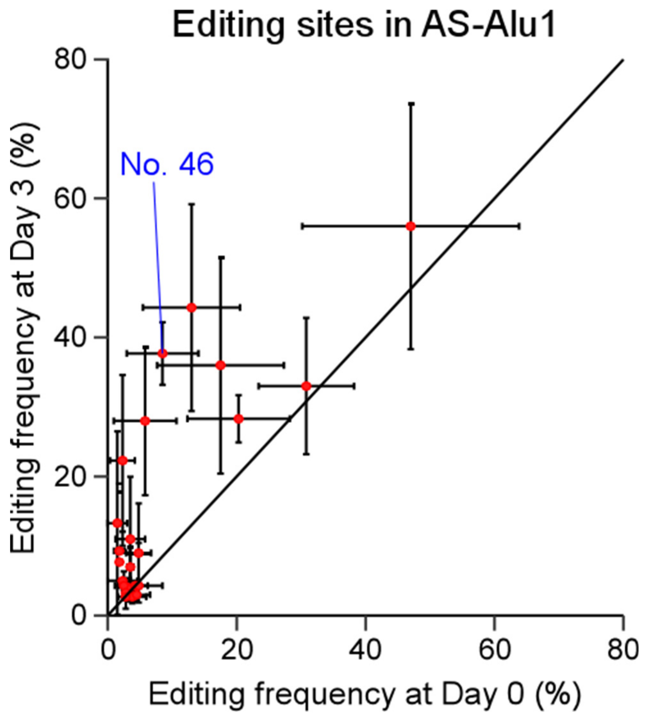

Supplementary Figure 12. Dynamic alteration of A-to-I RNA editing in AS-Alu1 of *SELENON* mRNA during Hu5/KD3 differentiation.

Comparison of the A-to-I RNA editing frequency in AS-Alu1 between Days 0 and 3. The frequency of each editing site in AS-Alu1 is plotted. Data are presented as mean values +/- S.D.; *n*=4 (Day 0) and *n*=3 (Day 3) biologically independent samples. Source data are provided in Supplementary Data 1.

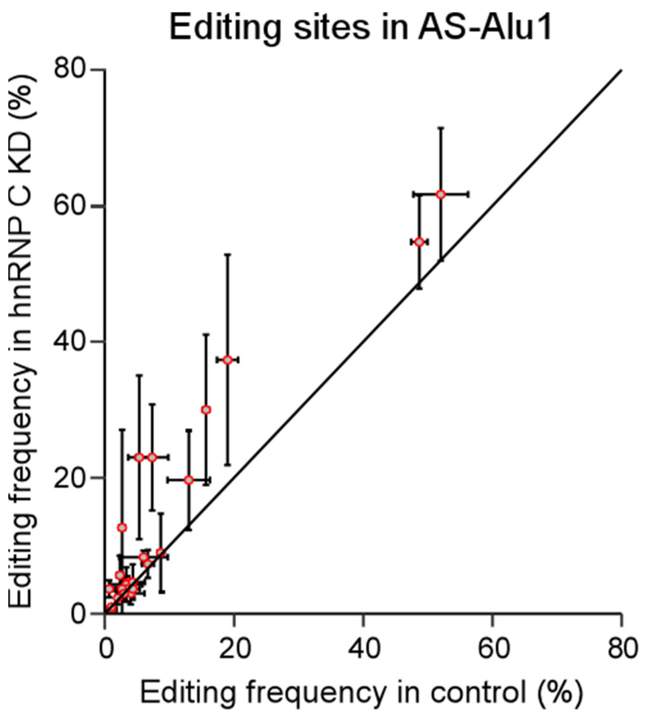

Supplementary Figure 13. Alteration of A-to-I RNA editing in AS-Alu1 of *SELENON* mRNA upon *hnRNP C* knock down.

Comparison of the A-to-I RNA editing frequency in AS-Alu1 of *SELENON* mRNA in HeLa cells treated with siRNAs targeting *luciferase* (control) and *hnRNP C*. Data are presented as mean values +/- S.D.; *n*=3 biologically independent samples. Source data are provided in Supplementary Data 1.

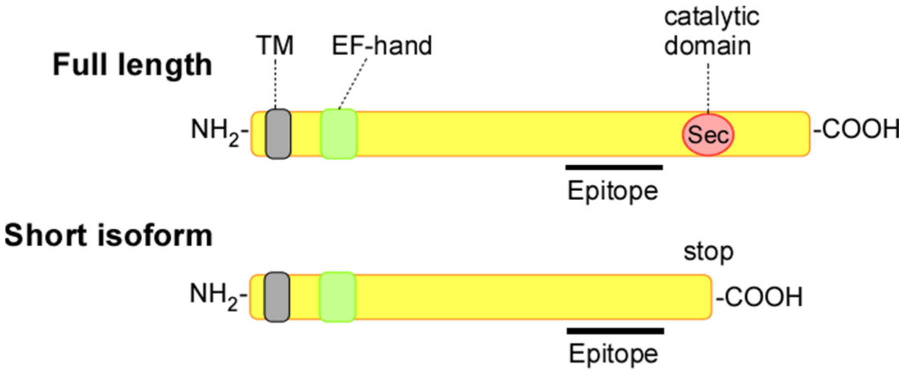

Supplementary Figure 14. Domain structure of human *SELENON*.
TM (transmembrane) and EF-hand domains are shown in both full-length and short isoforms. The Sec residue in the catalytic domain is indicated. The epitope for the anti-SELENON antibody is underlined.

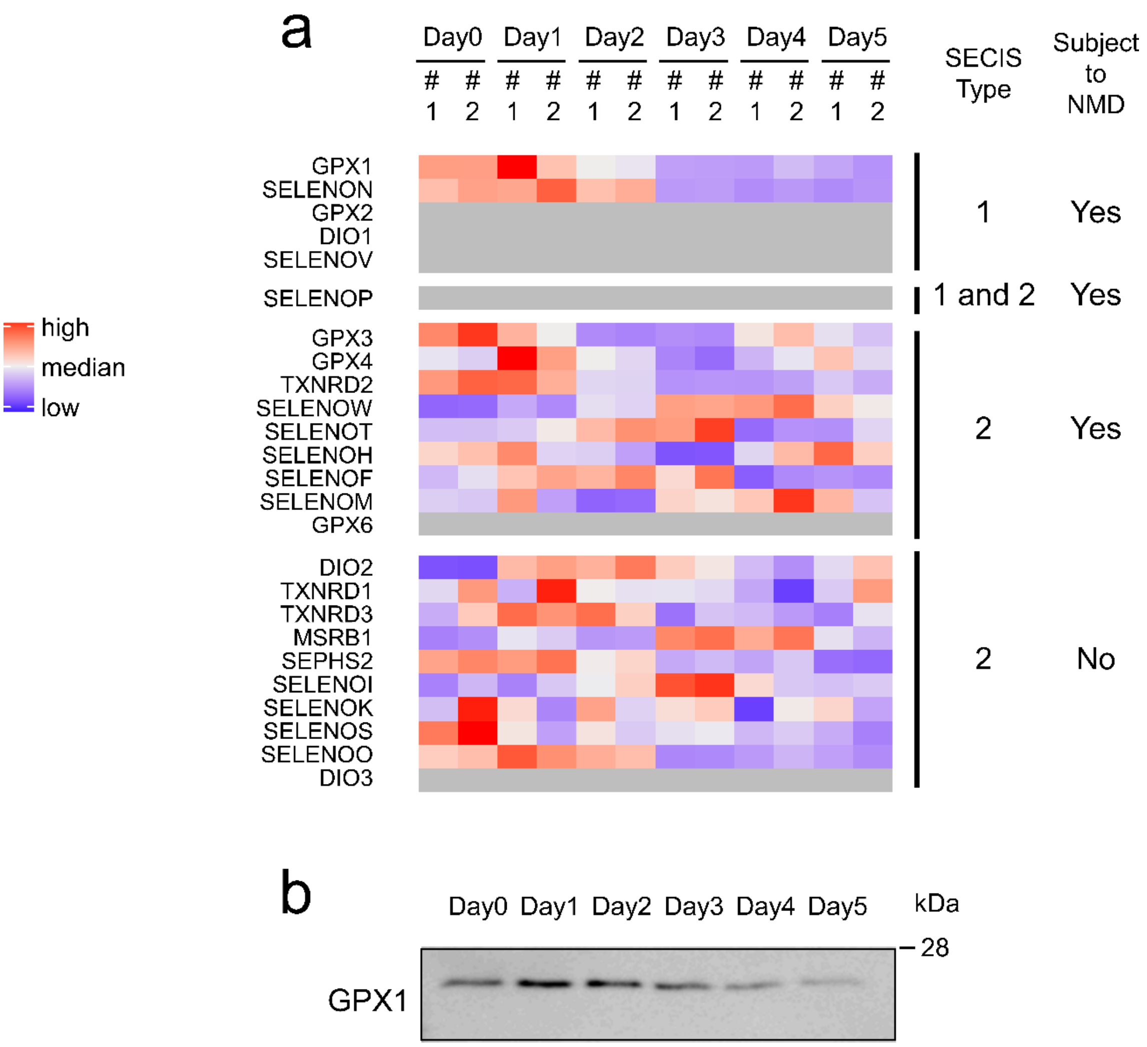

Supplementary Figure 15. Expression profiles of selenoproteins during myoblast differentiation.

(a) Heat map of RNA-seq transcriptome analysis of selenoproteins on each day of Hu5/KD3 differentiation. Colors correspond to the per-gene z-scores computed from $\log_{10}$(fragments per million reads mapped [FPM]). Selenoproteins are classified by SECIS type and NMD sensitivity. Source data are provided in Source Data file.

(b) Expression analysis of *GPX1* by western blotting on each day of Hu5/KD3 differentiation. This experiment was carried out once. Unprocessed gel image is provided in Source Data file.

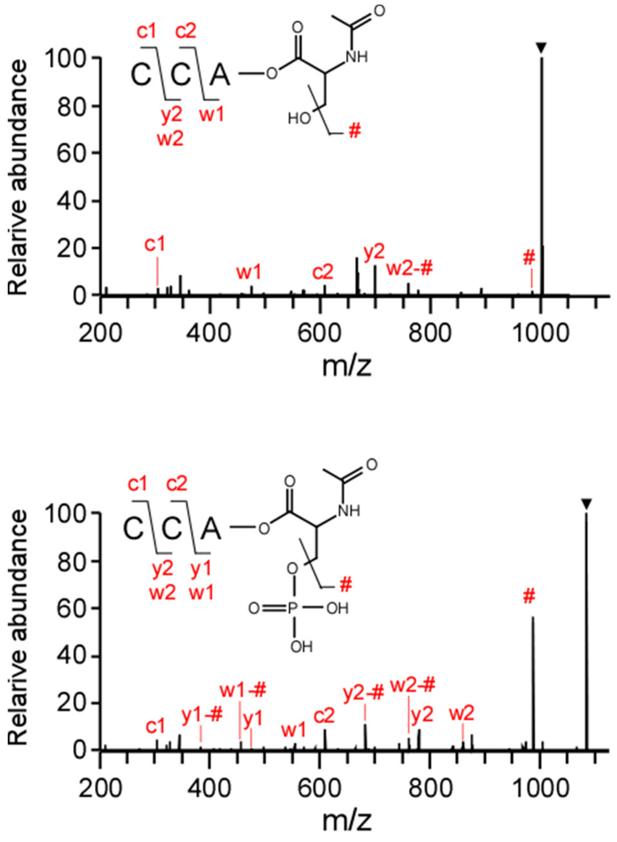

Supplementary Figure 16. Mass spectrometric analyses of amino acids attached to tRNA^{Sec}.

Collision-induced dissociation spectrum of the CCA trinucleotides with Ser (upper panel) and phosphoserine (pSer) (lower panel) bearing acetylation (Ac). Product ions are assigned as indicated on the sequence.

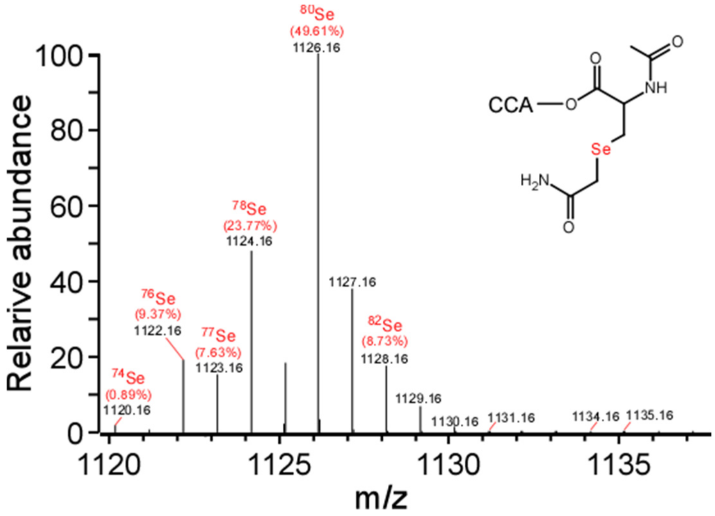

Supplementary Figure 17. Isotope simulation of CCA trinucleotide bearing Sec. Percentages of natural Se isotope distribution are depicted in red. The chemical structure of the molecule is shown on the panel.

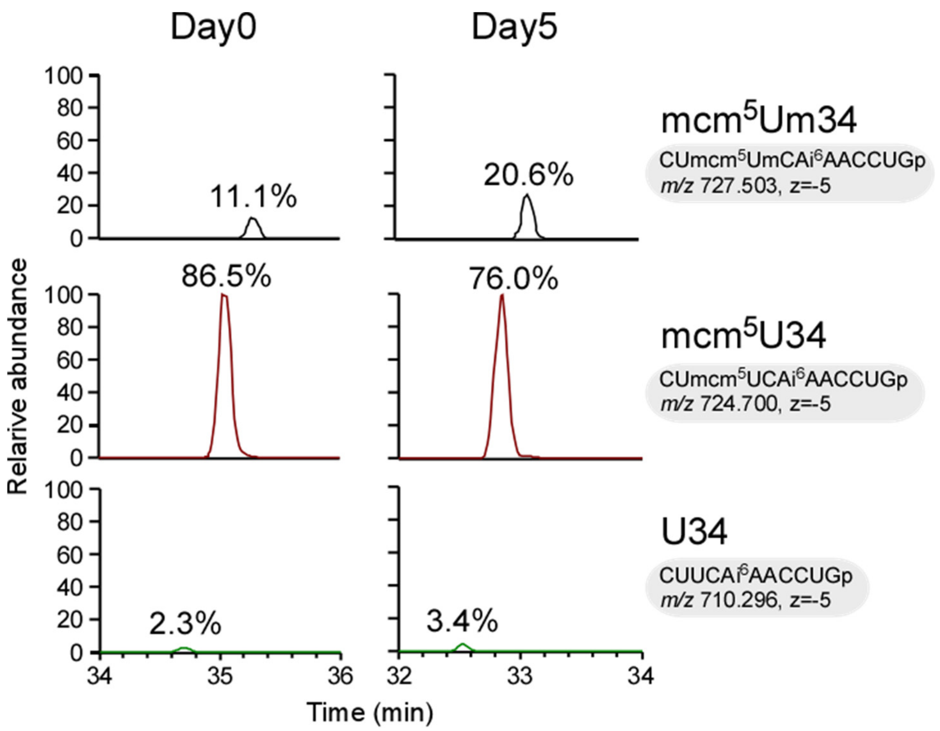

Supplementary Figure 18. Mass spectrometric analysis of tRNA^{Sec} modifications during myoblast differentiation.

XICs for the anticodon-containing fragments of tRNA^{Sec} isolated on Day 0 and 5 of Hu5/KD3 differentiation. The frequency of each modification is calculated from the relative peak intensities of the fragments bearing mcm^{5}Um, mcm^{5}U, or U in the anticodon. The tRNA modification, sequence, *m/z* value, and charge state are shown on the right.

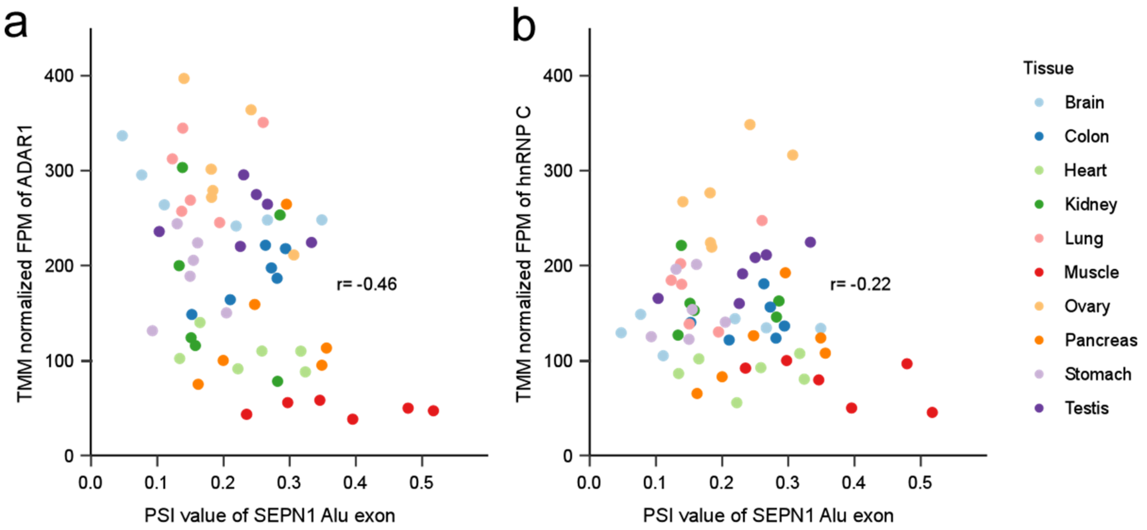

Supplementary Figure 19. Comparison of Alu exonization level versus steady-state level of *ADAR1* or *hnRNP C* in individual human tissues.

Correlation of percent splice-in (PSI) value of *SELENON* Alu exon and steady-state level of *ADAR1* (a) or *hnRNP C* (b) in the GTEx samples. Plot colors indicate tissue type, according to the legend on the right. Source data are provided in Supplementary Data 3.

Supplementary References

1 Carithers, L. J. *et al.* A Novel Approach to High-Quality Postmortem Tissue Procurement: The GTEx Project. *Biopreserv Biobank* **13**, 311-319, doi:10.1089/bio.2015.0032 (2015).

2 Sakurai, M., Yano, T., Kawabata, H., Ueda, H. & Suzuki, T. Inosine cyanoethylation identifies A-to-I RNA editing sites in the human transcriptome. *Nat Chem Biol* **6**, 733-740 (2010).

3 Zuker, M. Mfold web server for nucleic acid folding and hybridization prediction. *Nucleic Acids Res* **31**, 3406-3415, doi:10.1093/nar/gkg595 (2003).
