# Supplementary material for: Regulation of A-to-I RNA editing and stop codon recoding to control selenoprotein expression during skeletal myogenesis
Source: Nat Commun. 2022 May 6;13:2503. doi: 10.1038/s41467-022-30181-2 (PMC9076623; doi:10.1038/s41467-022-30181-2)
Supplement: Supplementary file 3 — Description of Additional Supplementary Files [file 41467_2022_30181_MOESM3_ESM.docx]

Description of Additional Supplementary Files

File Name: Supplementary Data 1-4

Description:

Supplementary Data 1. A-to-I RNA editing sites with their frequencies in the Alu elements of SELENON pre-mRNA

Supplementary Data 2. List of RNase T1-digested fragments of human tRNASec

Supplementary Data 3. List of GTEx human tissues for transcriptome analysis

Supplementary Data 4. List of primers, probes and siRNAs used in this study
