# Supplementary material for: Regulation of A-to-I RNA editing and stop codon recoding to control selenoprotein expression during skeletal myogenesis
Source: Nat Commun. 2022 May 6;13:2503. doi: 10.1038/s41467-022-30181-2 (PMC9076623; doi:10.1038/s41467-022-30181-2)
Supplement: Supplementary file 5 — Reporting Summary [file 41467_2022_30181_MOESM5_ESM.pdf]

nature research

Corresponding author(s): Tsutomu Suzuki

Last updated by author(s): Mar 3, 2022

Reporting Summary

Nature Research wishes to improve the reproducibility of the work that we publish. This form provides structure for consistency and transparency in reporting. For further information on Nature Research policies, see our Editorial Policies and the Editorial Policy Checklist.

Statistics

For all statistical analyses, confirm that the following items are present in the figure legend, table legend, main text, or Methods section.

n/a	Confirmed	
☐	☒	The exact sample size (*n*) for each experimental group/condition, given as a discrete number and unit of measurement
☐	☒	A statement on whether measurements were taken from distinct samples or whether the same sample was measured repeatedly
☐	☒	The statistical test(s) used AND whether they are one- or two-sided<br>*Only common tests should be described solely by name; describe more complex techniques in the Methods section.*
☒	☐	A description of all covariates tested
☒	☐	A description of any assumptions or corrections, such as tests of normality and adjustment for multiple comparisons
☐	☒	A full description of the statistical parameters including central tendency (e.g. means) or other basic estimates (e.g. regression coefficient) AND variation (e.g. standard deviation) or associated estimates of uncertainty (e.g. confidence intervals)
☐	☒	For null hypothesis testing, the test statistic (e.g. *F*, *t*, *r*) with confidence intervals, effect sizes, degrees of freedom and *P* value noted<br>*Give P values as exact values whenever suitable.*
☒	☐	For Bayesian analysis, information on the choice of priors and Markov chain Monte Carlo settings
☒	☐	For hierarchical and complex designs, identification of the appropriate level for tests and full reporting of outcomes
☒	☐	Estimates of effect sizes (e.g. Cohen's *d*, Pearson's *r*), indicating how they were calculated

Our web collection on statistics for biologists contains articles on many of the points above.

Software and code

Policy information about availability of computer code

Data collection	Gel images and Northern blotting image were obtained by FLA-7000.<br>RT-qPCR data were obtained by LightCycler480.<br>Western blotting images were obtained by ImageQuant LAS4000 mini.<br>RNA-Seq raw data was obtained by HiSeq 4000 (150-bp, single-read).<br>Immunostaining images were obtained by DMI 6000 B.<br>LC/MS data was obtained by LTQ Orbitrap XL.<br>Cell images were obtained by IncuCyte S3 Live-Cell Analysis System.
Data analysis	Canvas 11 and R 4.0.2 were used to draw figures and analyze statistical data.<br>CodonCode Aligner 2.0.6 was used to analyze sequencing chromatograms.<br>IGV 2.8.6 was used to map identified editing sites.<br>Muti Gauge V3.0 was used for graphical analysis.<br>Trimmomatic 0.36 was used for raw read quality control and adapter trimming.<br>Trimmed reads were mapped against hg38 human genome reference using STAR 2.5.3a.<br>Mapped counts were determined by htseq-count 0.9.1.<br>Differential expression analysis was performed using edgeR 3.32.1.<br>Xcalibur Qual browser was used for mass spec analysis.<br>The secondary structure of SEPN1 mRNA was predicted by Mfold.<br>The intensity of western blotting bands were analyzed using ImageJ 1.53K.

For manuscripts utilizing custom algorithms or software that are central to the research but not yet described in published literature, software must be made available to editors and reviewers. We strongly encourage code deposition in a community repository (e.g. GitHub). See the Nature Research guidelines for submitting code & software for further information.

Data

Policy information about availability of data

All manuscripts must include a data availability statement. This statement should provide the following information, where applicable:

- Accession codes, unique identifiers, or web links for publicly available datasets
- A list of figures that have associated raw data
- A description of any restrictions on data availability

The sequence data from this study have been submitted to the NCBI Sequence Read Archive under BioProject ID PRJNA705848.
The dbGaP GTEx Analysis V8 release is available under the accession phs00424.v8.p2; https://www.ncbi.nlm.nih.gov/projects/gap/cgi-bin/study.cgi?study_id=phs000424.v8.p2

Field-specific reporting

Please select the one below that is the best fit for your research. If you are not sure, read the appropriate sections before making your selection.

☒ Life sciences ☐ Behavioural & social sciences ☐ Ecological, evolutionary & environmental sciences

For a reference copy of the document with all sections, see nature.com/documents/nr-reporting-summary-flat.pdf

Life sciences study design

All studies must disclose on these points even when the disclosure is negative.

Sample size
Sample size of each experiment was described in the Figure Legends of the main text and supplementary information. Each sample size was determined according to previous studies in our lab or published papers performing similar experiments. No statistical method was used to pre-determine each sample size.

Data exclusions
In RNA-seq analysis, genes whose reads were not counted in any sample were excluded in further analyses. This exclusion criterion was not pre-established in this study, but generally employed in RNA-seq analysis.

Replication
Experiments in the minigene reporter analysis (Figure 1d), the expression analysis of SELENON WT and mutant constructs (Figure 4b), the subcellular localization analysis of SEPSECS (Figure 4f) and the analysis of tRNASec (Figure 5b,c,d,f,g) were performed one time in no replication. The other experiments were replicated in at least two biologically independent samples or independently performed at least two times with the same results.

Randomization
Randomization was not required in this study because samples were collected from the heterogeneous group of cells.

Blinding
Blinding was not required in this study because it would not increase the reliability of the results.

Reporting for specific materials, systems and methods

We require information from authors about some types of materials, experimental systems and methods used in many studies. Here, indicate whether each material, system or method listed is relevant to your study. If you are not sure if a list item applies to your research, read the appropriate section before selecting a response.

Materials & experimental systems

n/a	Involved in the study	
☐	☒	Antibodies
☐	☒	Eukaryotic cell lines
☒	☐	Palaeontology and archaeology
☒	☐	Animals and other organisms
☒	☐	Human research participants
☒	☐	Clinical data
☒	☐	Dual use research of concern

Methods

n/a	Involved in the study	
☒	☐	ChIP-seq
☒	☐	Flow cytometry
☒	☐	MRI-based neuroimaging

Antibodies

Antibodies used
anti-ADAR1 (sc-271854, Santa Cruz Biotechnology), anti-ADAR2 (sc-73409, Santa Cruz Biotechnology), anti-DYKDDDDK (FLAG) (014-22383, Wako), anti-GAPDH (AM4300, Invitrogen), anti-GPX1 (sc-133160, Santa Cruz Biotechnology), anti-hnRNP C1/C2 (sc-32308, Santa Cruz Biotechnology), anti-MYOG (sc-576, Santa Cruz Biotechnology), anti-MYH1 (MF20-S, DSHB), anti-SELENON (sc-365824, Santa Cruz Biotechnology), anti-SEPSECS (A7103, ABclonal), anti-RENT1 (UPF1) (A300-038A, BETHYL), Peroxidase affinipure donkey anti-mouse/rabbit IgG (715-035-150/715-035-152, Jackson ImmunoResearch), peroxidase affinipure bovine anti-

goat IgG (805-035-180, Jackson ImmunoResearch), anti-mouse/rabbit IgG Alexa fluor 488 (A11029/A11008, Invitrogen), anti-mouse IgG Alexa fluor 594 (A11005, Invitrogen)

Validation

According to the manufacturer's websites, antibodies were validated to react with human proteins by western blotting analysis. anti-ADAR1, anti-ADAR2, anti-GAPDH, anti-GPX1, anti-hnRNP C1/C2, anti-MYOG, anti-MYH1, anti-SELENON, anti-RENT1 (UPF1)

According to the manufacturer's website, an antibody was validated to react with human protein by western blotting and immunofluorescence analysis.
anti-SEPSECS

According to the manufacture's website, an antibody was validated to react with a FLAG-tagged protein by western blotting analysis.
anti-DYKDDDDK (FLAG)

Eukaryotic cell lines

Policy information about cell lines

Cell line source(s)

HeLa and HEK293T cells were purchased from ATCC. Hu5/KD3 cells were kindly provided by Dr. Naohiro Hashimoto (National Center for Geriatrics and Gerontology, Japan).

Authentication

None of the cell lines used were authenticated.

Mycoplasma contamination

The cell lines were not checked for mycoplasma contamination.

Commonly misidentified lines
(See ICLAC register)

No commonly misidentified cell lines used.
